# Myocardial Injury in Multiple Myeloma Patients With Preserved Left Ventricular Ejection Fraction: Noninvasive Left Ventricular Pressure-Strain Myocardial Work

**DOI:** 10.3389/fcvm.2021.782580

**Published:** 2022-01-20

**Authors:** Zhiyue Liu, Li Zhang, Mei Liu, Fang Wang, Yanqiu Xiong, Zhuoqin Tang, Qian Li, Qiuchen Lu, Shichu Liang, Ting Niu, He Huang

**Affiliations:** ^1^Department of Cardiology, West China Hospital, Sichuan University, Chengdu, China; ^2^Department of Hematology, West China Hospital, Sichuan University, Chengdu, China; ^3^Department of Ultrasound, Mianyang Central Hospital, Mianyang, China

**Keywords:** cardiac injury, multiple myeloma, cardiac adverse events, preserved left ventricular ejection fraction, left ventricular pressure-strain-derived myocardial work

## Abstract

**Introduction:**

Over one-half of patients with multiple myeloma (MM) die of heart failure or arrhythmia. Left ventricular ejection fraction (LVEF) is used to describe left ventricular systolic function. However, depressed LVEF means advanced stage of left ventricular dysfunction in patients with MM. Left ventricular pressure-strain-derived myocardial work (LVMW) is a novel and noninvasive method for evaluating LV function related to LV dynamic pressure load. MW is assessed by LV MW index (LVMWI), constructive work, wasted work, and LV MW efficiency (LVMWE). In this study, we aimed to investigate the value of LVMW in cardiac function assessment and clinical prognosis of MM patients with preserved LVEF.

**Methods:**

A total of 72 subjects, including 40 untreated MM patients with preserved EF (including the thick wall and normal wall groups) and 32 non-MM patients, were enrolled in this study. Laboratory data and clinical history of all the patients were collected. All the patients underwent comprehensive echocardiographic examinations and then LVMWI and LVMWE were calculated. Moreover, cardiac adverse events (CAEs) were observed in MM patients treated with bortezomib-based therapy after 6 months and the prognostic value of MW was assessed.

**Results:**

(1) LV myocardial global work index (GWI), myocardial global work efficiency (GWE), and global longitudinal strain (GLS) were lower in the thick wall group of patients with MM compared with the normal wall group and controls. Cardiac segmental analysis of LVMWI in patients with MM showed an apical sparing pattern; (2) The area under the curve (AUC) of GWE for judging the disease severity based on the Revised International Staging System (R-ISS) was 0.835 (95% CI: 0.684–0.933, *p* < 0.05); (3) GWE, Lg_dFLC_, and arrhythmia were independent risk factors of CAEs. The AUC of GWE for predicting CAEs in MM patients treated with bortezomib-based therapy for 6 months follow-up was 0.896 (95% CI: 0.758–0.970, *p* < 0.05).

**Conclusion:**

MM Patients with preserved EF had subclinical LV systolic dysfunction, which was worse in the thick wall group. LVMWI was presented as “apical sparing” in patients with MM. A lower LVGWE may have a predictive value for CAEs in patients with MM after 6 months of follow-up.

## Introduction

Multiple myeloma (MM) is a multiple-system disease with the overproduction of monoclonal immunoglobulins and clonal proliferation of neoplastic plasma cells in the elderly (1). Meanwhile, proteasome inhibitors such as Bortezomib and Carfilzomib are an essential part of the treatment of MM, which might lead to cardiotoxicity through the protein aggregation and alter transcriptional activation of NF-κB targets in cardiomyocytes (2, 3). Cardiac involvement remains a critical determinant of prognosis regardless of age (4). Over one-half of patients with MM die of heart failure or arrhythmia. The median survival time of patients with MM has decreased to 6 months when heart failure was present (5, 6). Left ventricular ejection fraction (LVEF) is used to describe left ventricular function, but depressed LVEF means the advanced stage of left ventricular dysfunction. A new parameter to detect early cardiac dysfunction is necessary.

Left ventricular global longitudinal strain (LVGLS) has proven to be reliable for both the diagnosis and risk stratification in patients with cardiac dysfunction (7), especially in those with preserved LVEF (8). As previously reported, in patients undergoing chemotherapy, changes in GLS were found earlier than LVEF changes, which is of help to detect cardiotoxicity, with a 91% sensitivity and 83% specificity (9). Two-dimensional (2D) speckle-tracking imaging showed that cardiac injury in patients with MM is characterized by reduced basal strain (10), which suggests an early LV systolic dysfunction. However, the strain does not take into consideration LV afterload. Left ventricular myocardial work index (LVMWI) is a novel and noninvasive method for LV work analysis (11). The combination of LV deformation and afterload by constructing an LV pressure-strain loop (PSL) integrated measured arterial blood pressure and longitudinal strain (LS) acquired by echocardiographic speckle-tracking analysis (12).

In this study, we aimed to investigate the value of left ventricular pressure-strain-derived myocardial work (LVMW) in cardiac function and clinical prognosis in MM patients with preserved LVEF. This may assist clinicians in the early detection of myocardial injury.

## Materials and Methods

### Study Population

**Figure 1:**
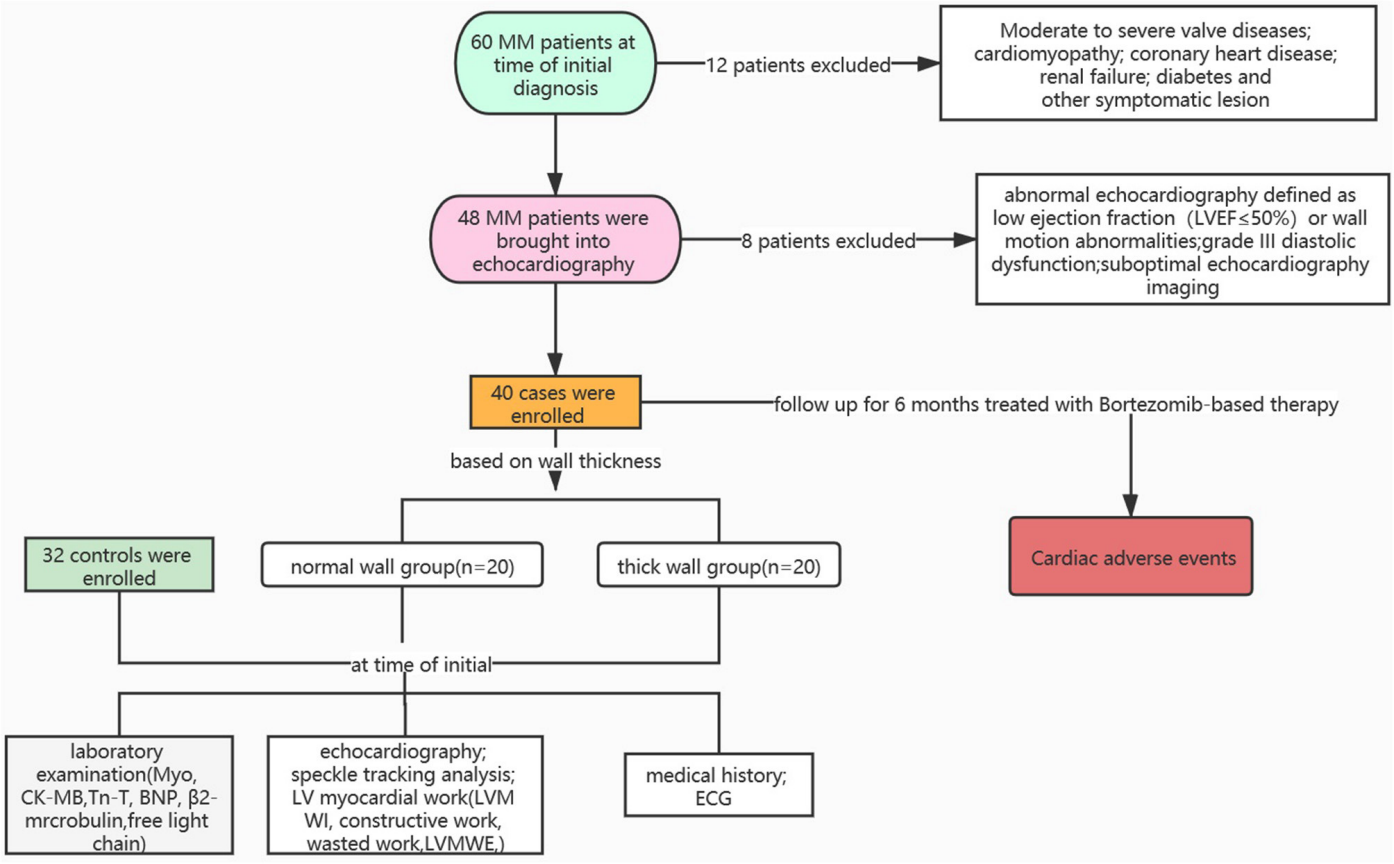
Overview of patient selection process. Myo, myoglobin; CK-MB, creatine kinase-MB; TnT, troponin T; BNP, brain natriuretic peptide.

A total of 60 patients with MM were recruited at the time of initial diagnosis between January 1, 2020 and January 1, 2021 at West China Hospital, Sichuan University. The process for selecting eligible patients is shown in Figure 1. Inclusion criteria were age >18 years, diagnosis of symptomatic MM according to 2013 WHO diagnostic criteria, and disease severity was staged according to the Revised International Staging System (R-ISS) based on baseline β2 macroglobulin (β2M) and serum albumin levels. Exclusion criteria were abnormal echocardiography (defined as LVEF ≤ 50%), wall motion abnormalities, moderate-to-severe valvular disease or high-grade diastolic dysfunction (grade III diastolic dysfunction: mitral *E*/*A* ratio >2 or average *E*/*e*′ ratio > 14), coronary heart disease, cardiomyopathy, renal failure, or other significant alterations. In total, 40 eligible patients with MM were divided into the two groups according to the thickness of the LV wall (thick wall group was defined as wall thickness >10 mm in female patients or >11 mm in male patients): the normal wall group (*n* = 20) and the thick wall group (*n* = 20). In total, 32 non-MM patients who had normal echocardiography and matched with age, gender, and blood pressure were selected as the control group. Clinical history and laboratory examination of patients with MM were collected. All the patients with MM were stratified based on the R-ISS (13). The R-ISS stage I: serum β2M level was < 3.5 mg/l and serum albumin was ≤ 3.5 g/dl, no high-risk cytogenetic abnormality (CA) [del(17p) and/or t(4;14) and/or t(14;16)], and normal lactic dehydrogenase level; the R-ISS stage III: serum β2M level > 5.5 mg/l and high-risk CA or high lactic dehydrogenase level; and the R-ISS II: including all the other possible causes. All the procedures were approved by the Biomedical Research Ethics Committee of West China Hospital, Sichuan University and written informed consent was obtained from all the study participants.

### Serological Indicators of Patients With MM

Fasting venous blood samples were collected. Monoclonal (M) protein was detected by serum immunoelectrophoresis with an automatic electrophoresis analyzer and its supporting reagent (Serbia Hydras, France). A serum-free light chain (FLC) kit (Binding Site, England, UK) was used to determine the serum-FLC level. Referring to the type of involved monoclonal FLC, which was kappa or lambda FLC, the difference between the involved FLC and uninvolved FLC was defined as dFLC. The serum β2M level was detected using a scattering immune turbidimetry automatic protein analyzer (Siemens, Germany).

### Echocardiography

The ultrasound system (Vivid E95; GE Vingmed Ultrasound, Horten, Norway) with a 1.7–3.3 MHz phased-array transducer (M5S) was used.

### Standard Echocardiographic Examination

Standard echocardiography, including two-dimensional (2D), M-mode, and Doppler echocardiography, was performed according to the guidelines of the American Society of Echocardiography (14). LV end-diastolic and end-systolic volumes and LVEF were measured based on the modified biplane Simpson's rule. Mitral inflow velocity at early (E) and late (A) diastole were measured. The velocity of the mitral annulus at early diastolic (e′) and late diastolic (a′) myocardial were recorded by pulsed tissue Doppler imaging. The E/e′ ratio was used as an index of LV diastolic function. All the images were captured by a senior operator.

### Pressure-Strain-Derived MW

Myocardial work was calculated using a PSL curve integrated with LV deformation and pressure. Deformation was measured as LS by the speckle-tracking technique. Peak systolic LV pressure was assumed to be equal to the peak arterial pressure, which was measured immediately before the echocardiographic study using an arm-type mercury sphygmomanometer. Then, a noninvasive LV pressure curve adjusted according to the duration of isovolumic and ejection phases defined by valvular timing events was constructed.

Image acquisition: Dynamic images were collected in three planes: apical four-, two-, and three-chamber planes for more than 3 cardiac cycles. Then, the data were analyzed offline by EchoPAC 203 workstation (Vivid E95; GE Vingmed Ultrasound, Horten, Norway). Initially, the myocardial automatic functional imaging analysis mode was selected. The system could automatically recognize the above three dynamic images and select the cardiac cycle with the best image quality for myocardial tracking of the motion trajectory. If there is a deviation in the tracking, the position and size of the area of interest can be manually adjusted. Initial tracking was conducted at the apical three-chamber to confirm the closing time of the aortic valve and other planes have completed the analysis in turn. Then, the system generated a 17-segment bull's-eye automatically, which is obtained according to the weighted average of the peak LS of each segment during systole. The overall global longitudinal strain (GLS) was expressed in absolute values. Finally, the MW analysis mode was selected to analyze and obtain LV-PSL.

**Figure 2:**
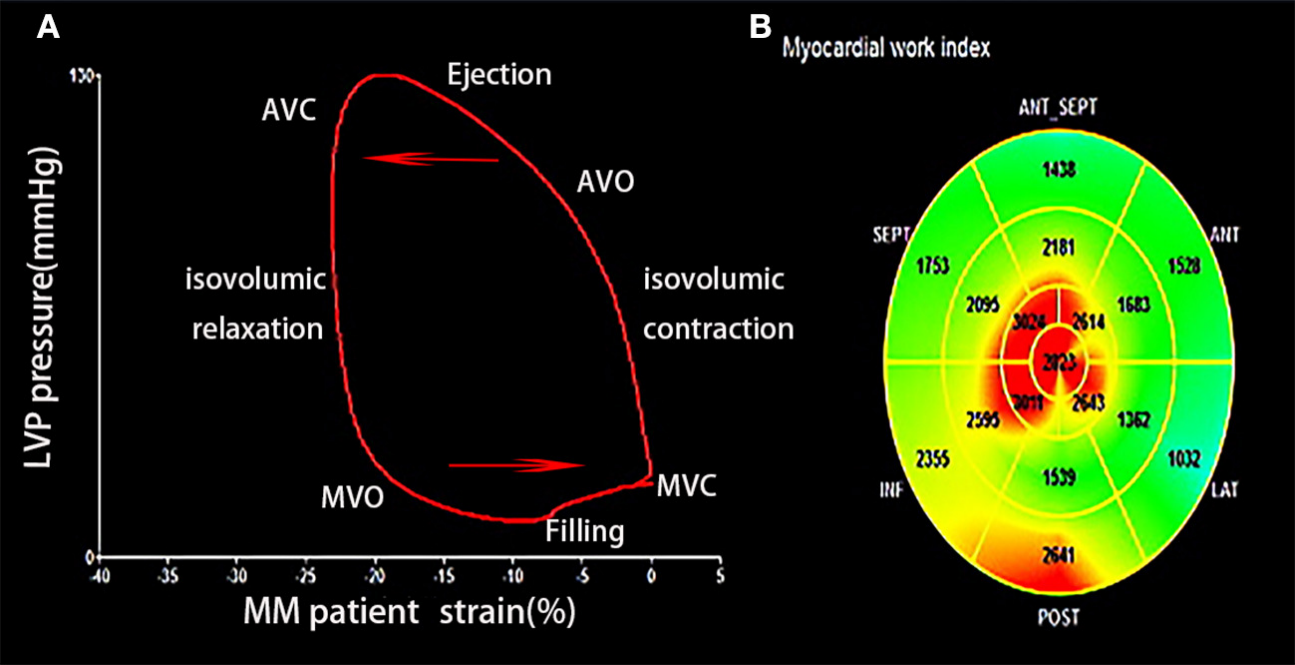
**(A)** Left ventricular (LV) pressure-strain loops of patient with MM showing LV pressure and GLS change during the cardiac cycle. **(B)** Segmental GWI of LV. MM, multiple myeloma; GLS, global longitudinal strain; GWI, global work index; AVC, aortic valve closure; AVO, aortic valve opening; MVC, mitral valve closure; MVO, mitral valve opening.

Characteristics of MW in patients with MM are shown in Figure 2. LV work index and efficiency of all the segmental values were averaged. The area within the PSL provided the MW index (WI). The following parameters were calculated (11):

(1) Global WI (GWI): Total work within the area of the LV-PSL calculated from mitral valve opening and closure.
(2) Constructive myocardial work: Work contributing to LV ejection during systole.
  constructive MW = The area of (the peak arterial pressure × the strain of myocytes shorting during systole and relaxation during isovolumic period)
  constructive MW = The area of (the peak arterial pressure × the strain of myocytes shorting during systole and relaxation during isovolumic period)
(3) Wasted myocardial work: Work performed by the LV that does not contribute to LV ejection.
  Wasted myocardial work = The area of (the peak aterial pressure × the strain of myocytes lengthening during systole and shorting during isovolumic period)
(4) Myocardial work efficiency: The ratio of work contributing to LV ejection and total work.
  Myocardial work efficiency = Constructive myocardial work / (constructive myocardial work + wasted myocardial work)

### Follow-Up

All the patients were followed up after 6 months for their survival and cardiac adverse events (CAEs). All the patients received bortezomib-based therapy. CAEs were defined following the recommendations in the common terminology criteria for adverse events version 4.0 (15). Cardiac disorders include acute coronary syndrome, valve disease, asystole, cardiac arrest, chest pain, heart failure, left and right ventricular systolic dysfunction, myocarditis, myocardial infarction, palpitations, arrhythmia, and pericarditis.

### Statistical Analysis

Continuous variables were presented as mean ± SD for normally distributed data or median (25th percentile and 75th percentile) for nonnormally distributed data. Categorical variables were presented as frequencies. The *t*-test and one-way ANOVA were adopted for comparison of two and three independent groups of normally distributed variables, respectively. The Wilcoxon signed-rank test was used for nonnormal distribution. The chi-squared test and Fisher's exact test were used to compare binary variables. The Spearman and Pearson correlation coefficients were calculated for dFLC and other MM-related parameters and N-terminal probrain natriuretic peptide (NT-proBNP). The multivariable logistic regression models were used to further assess the risk factors of CAEs. The univariate regression analysis of variables, positive variables, and important clinically significant indicators were included in the multivariate regression analysis model. The receiver operating characteristic (ROC) curve analysis was performed adjusting for NT-proBNP and echocardiographic indices. Value of *p* < 0.05 was considered statistically significant. IBM SPSS for Windows version 17.0 (IBM Corporation, Armonk, New York, USA) was used for all the analyses.

## Results

### Clinical Characteristics

The clinical characteristics of 40 patients with MM and 32 control subjects are shown in Table 1. There were no differences in age, sex, body mass index (BMI), and blood pressure between MM patients and control subjects. Patients with MM had a higher heart rate (HR) and mortality of arrhythmia, including atrial fibrillation, tachycardia, and high-degree atrioventricular (AV) block. The New York Heart Association (NYHA) functional class was higher in patients with MM than in controls. There were no differences in age, systolic blood pressure (SBP), HR, the mortality of arrhythmia, and the NYHA functional class between the normal wall group and the thick wall group of patients with MM. However, the thick wall group in patients with MM had a higher level of β2M, dFLC, myoglobin (Myo), creatine kinase-MB (CK-MB), troponin T (TnT), and NT-proBNP (Table 1).

**Table 1:** Characteristics of the study population.

	**Controls** **(*n =* 32)**	**Normal wall** **(*n =* 20)**	**Thick wall** **(*n =* 20)**	***P*-value**
Age, years	55.94 ± 7.10	59.55 ± 10.46	61.35 ± 10.66	0.103
Sex (male)	12 (37.5%)	10 (50%)	12 (60%)	0.673
BMI, kg/m^2^	23.19 ± 2.64	22.00 ± 3.29	23.15 ± 2.96	0.314
NYHA I/II/III/IV	32/0/0/0	18/2/0/0	15/3/2/0^*^	0.034
SBP, mmHg	125 ± 11	126 ± 18	130 ± 17	0.483
HR, bpm	70.39 ± 8.94	89.55 ± 22.21^*^	85.48 ± 17.49^*^	<0.001
Arrhythmias	0 (0)	3 (15%)^*^	7 (35%)^*^	0.002
Atrial fibrillation	0 (0)	1 (5%)	4 (20%)	0.061
tachycardia	0 (0)	2 (10%)	1 (5%)	0.249
A-V block	0 (0)	0 (0)	2 (10%)	0.499
Course, months	/	12.00 (5.25–33.00)	10.00 (4.00–33.25)	0.714
M protein (%)	/	18 (90%)	18 (90%)	1
β2M, ng/ml	/	4.20 (2.39–6.30)	9.76 (3.56–14.90)^#^	0.021
dFLC, ng/ml	/	140.54 (38.25–200.73)	510.50 (292.50–824.10)^#^	<0.001
Myo, ng/ml	/	23.29 (21.00–37.17)	68.70 (29.45–279.30)^#^	0.009
CK-MB, ng/ml	/	0.82 (0.56–1.74)	2.44 (1.22–9.85)^#^	0.003
Tn-T, ng/ml	/	13.25 (10.30–36.80)	63.00 (10.00–325.60)^#^	0.017
NT-pro BNP, ng/ml	/	350.00 (250.50–2,037.75)	6,928.00 (830.00–35,000.00)^#^	0.001

*BMI, Body mass index; SBP, systolic blood pressure; DBP, diastolic blood pressure; β2M, β2 microglobulin; dFLC, difference between involved and uninvolved free light chain; Myo, myoglobin; CK-MB, creatine kinase-MB; TnT, troponin T; BNP, brain natriuretic peptide*.

*Data are expressed as absolute number (percentage), mean ± SD, and median [interquartile range (IQR)]*.

* *Compared with controls, p < 0.05;*

# *Compared with the normal wall group, p < 0.05*.

### Standard Echocardiographic Characteristics

Conventional 2D and Doppler echocardiographic characteristics are shown in Table 2. There was no difference between MM patients and control subjects in the left atrium (LA), left ventricle (LV), right atrium (RA), right ventricle (RV), aortic root (AO) diameter, and LVEF. However, the basal segment of the interventricular septum (IVS) was thicker in the thick wall group of patients with MM than in the normal wall group and controls. The *E*/*e*′ ratio, an index of LV diastolic function, was higher in the thick wall group of patients with MM than in the normal wall group and controls. Moreover, the left ventricular mass index (LVMI) was higher in the thick wall group compared with the normal wall group and controls (*p* < 0.05). However, in a tricuspid regurgitation shown in 25 patients with MM, there was no statistical difference between the thick wall and normal wall groups.

**Table 2:** Standard echocardiographic characteristics of the study population.

	**Controls** **(*n =* 32)**	**Normal wall** **(*n =* 20)**	**Thick wall** **(*n =* 20)**	***P*-value**
LV, mm	45.60 ± 3.71	46.13 ± 4.94	46.93 ± 4.18	0.653
RV, mm	20.65 ± 1.79	20 ± 3.38	19.36 ± 2.24	0.281
LA, mm	29.70 ± 3.40	32.13 ± 6.85	31.43 ± 4.05	0.338
RA, mm	32.90 ± 2.90	32.63 ± 8.12	34.50 ± 5.33	0.595
IVS-basal, mm	7.90 ± 0.91	7.88 ± 0.64	12.93 ± 1.86^*^^#^	<0.001
AO, mm	29.55 ± 2.91	30.50 ± 5.81	28.71 ± 4.51	0.618
LVEF, %	61.65 ± 5.90	65.25 ± 2.71	59.71 ± 17.49	0.528
*E* wave, m/s	0.76 ± 0.17	0.61 ± 0.10	0.73 ± 0.15	0.075
*e*′, cm/s	9.45 ± 4.02	6.50 ± 2.33	5.93 ± 2.34^#^	0.008
*E*/*e*′	9.00 ± 2.88	10.28 ± 3.49	13.86 ± 6.46^*^	0.012
LVMI, g/m^2^	89.91 ± 12.01	96.45 ± 14.20	105.45 ± 11.95^*^^#^	<0.001

*LV, left ventricle; RV, right ventricle; LA, left atrium; RA, right atrium; IVS, interventricular septum; LVPW, left ventricular posterior wall; AO, aortic root; LVEF, left ventricular ejection fraction; E wave, early diastolic mitral peak inflow velocity; e′, early diastolic mitral annular velocity; LVMI, left ventricular mass index*.

*Data are expressed as absolute number (percentage) and mean ± SD*.

* *Compared with controls, p < 0.05;*

# *Compared with the normal wall group, p < 0.05*.

### Global and Segmental MW of the Left Ventricle in Patients With MM and Controls

As shown in Table 3, left ventricular global WI (GWI), global work efficiency (GWE), and GLS were lower in the thick wall group of patients with MM than in the normal wall group and controls (*p* < 0.05). Nevertheless, there was no statistical difference in GWI and GLS between the normal wall group of MM patients and controls. GWE was lower in the normal wall group than in controls. For segmental changes, we found that WI-basal was lower in the thick wall group than in the normal wall group and controls. WI-mid was higher in the normal wall group compared with the thick wall group and controls, while there was no difference in WI-mid between the thick wall group and controls. However, there was no significant difference in WI-apical among the three groups, which showed an apical sparing pattern.

**Table 3:** Longitudinal strain and myocardial work index of the study population.

	**Controls**	**Normal wall group**	**Thick wall group**	***P*-value**
	**(*n =* 32)**	**(*n =* 20)**	**(*n =* 20)**	
GLS, %	18.59 ± 2.37	17.23 ± 3.01	16.40 ± 2.95^*^	0.018
GWI, mmHg%	1643.38 ± 242.60	1756.25 ± 426.58	1450.08 ± 255.17^*^^#^	0.008
WI-basal, mmHg%	1517.55 ± 248.80	1582.77 ± 433.74	1298.41 ± 334.14^*^^#^	0.020
WI-mid, mmHg%	1548.81 ± 243.58	1751.48 ± 399.45^*^	1427.29 ± 200.38^#^	0.002
WI-apical, mmHg%	1863.79 ± 564.52	1934.51 ± 540.65	1624.54 ± 382.16	0.135
GWE, %	93.69 ± 2.89	91.50 ± 3.74*	87.75 ± 3.13^*^^#^	<0.001

*GLS, global longitudinal strain; WI, myocardial index; GWI, global WI; GWE, global work efficiency*.

*Data are expressed as mean ± SD*.

* *Compared with controls, p < 0.05;*

# *Compared with the normal wall group, p < 0.05*.

### Correlations Between LV Global Myocardial Work and Cardiotoxicities

**Figure 3:**
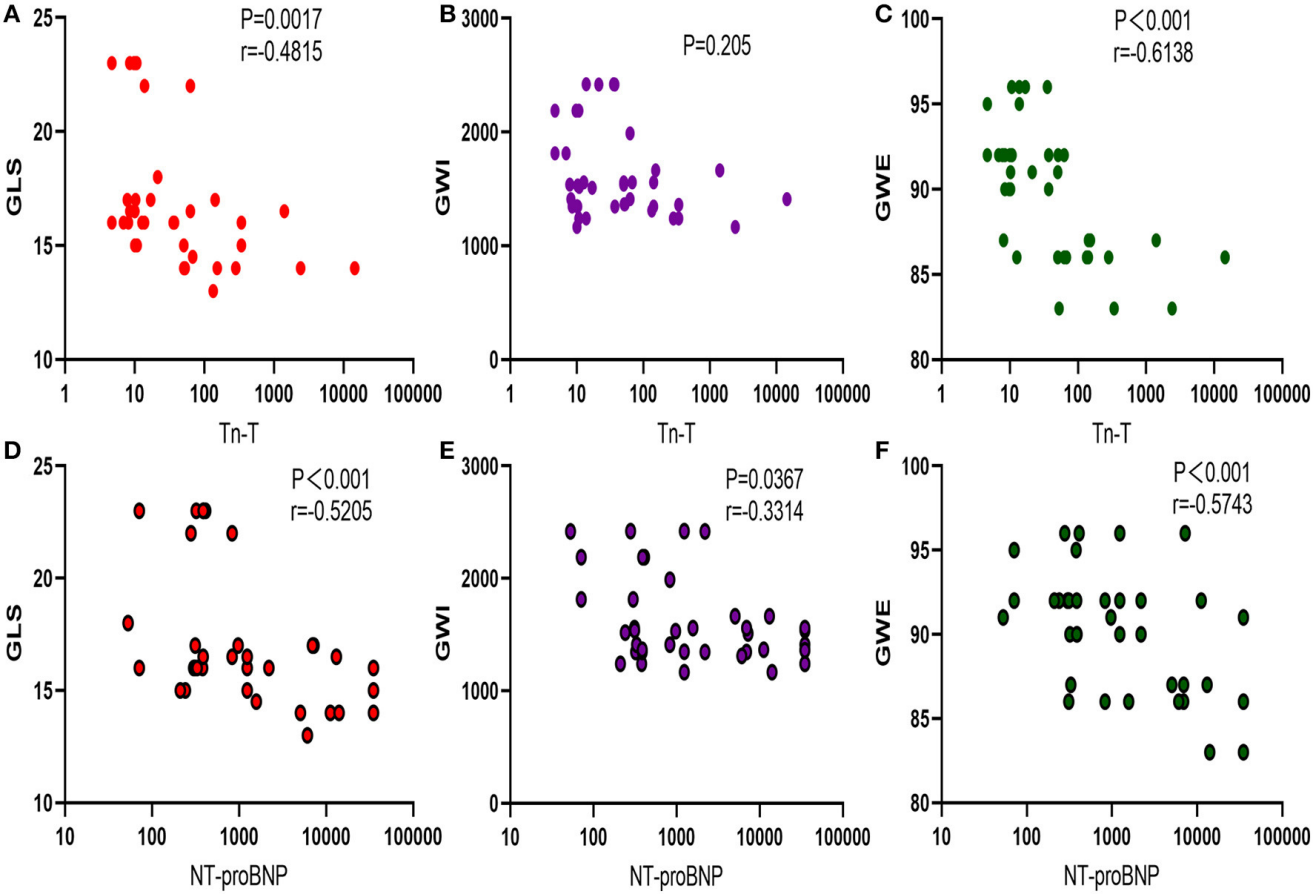
Correlation between myocardial injury biomarker and myocardial work. **(A–C)** Correlation between Tn-T and GLS, GWI, and GWE. **(D–F)** Negative correlation between NT-proBNP with GLS, GWI, and GWE. TnT, troponin T; GLS, global longitudinal strain; GWI, global work index; GWE, global work efficiency; NT-proBNP, N-terminal probrain natriuretic peptide.

Troponin T showed negative correlations with LV GLS (*p* < 0.05) and LV GWE (*p* < 0.05), but LV GWI (*p* > 0.05). NT-proBNP was negatively correlated with LV GLS, LV GWI, and LV GWE (*p* < 0.05) (Figure 3).

### Correlation Between the R-ISS of MM Patients and MW GLS

As shown in Table 4, the level of dFLC was positively correlated with TnT (*p* < 0.05), NT-proBNP (*p* < 0.05), and LVMI (*p* < 0.05), but negatively correlated with GLS (*p* < 0.05), GWI (*p* < 0.05), and GWE (*p* < 0.05). β2M, another indicator of MM disease severity, was positively correlated with NT-proBNP (*p* < 0.05) and negatively correlated with GLS (*p* < 0.05), GWI (*p* < 0.05), and GWE (*p* < 0.05).

**Table 4:** Correlation between MM biomarkers and cardiac function.

		**Tn-T (ng/ml)**	**NT-pro BNP (ng/ml)**	**GLS (%)**	**GWI (mmHg%)**	**GWE (%)**	**LVMI (g/m^**2**^)**
β2M, ng/ml	*r*	0.267	0.477^*^	−0.397^*^	−0.347^*^	−0.320^*^	0.237
	*P*-value	0.096	0.002	0.011	0.028	0.044	0.140
dFLC, ng/ml	*r*	0.583^*^	0.607^*^	−0.645^*^	−0.615^*^	−0.804^*^	0.688^*^
	*P*-value	0.005	<0.001	0.001	<0.001	<0.001	<0.001

*MM, multiple myeloma; dFLC, difference between involved and uninvolved free light chain; TnT, troponin T; BNP, brain natriuretic peptide; GLS, global longitudinal strain; LVMI, LV mass index; GWI, global work index; GWE, global work efficiency*.

* *A significant correlation, p < 0.05*.

**Figure 4:**
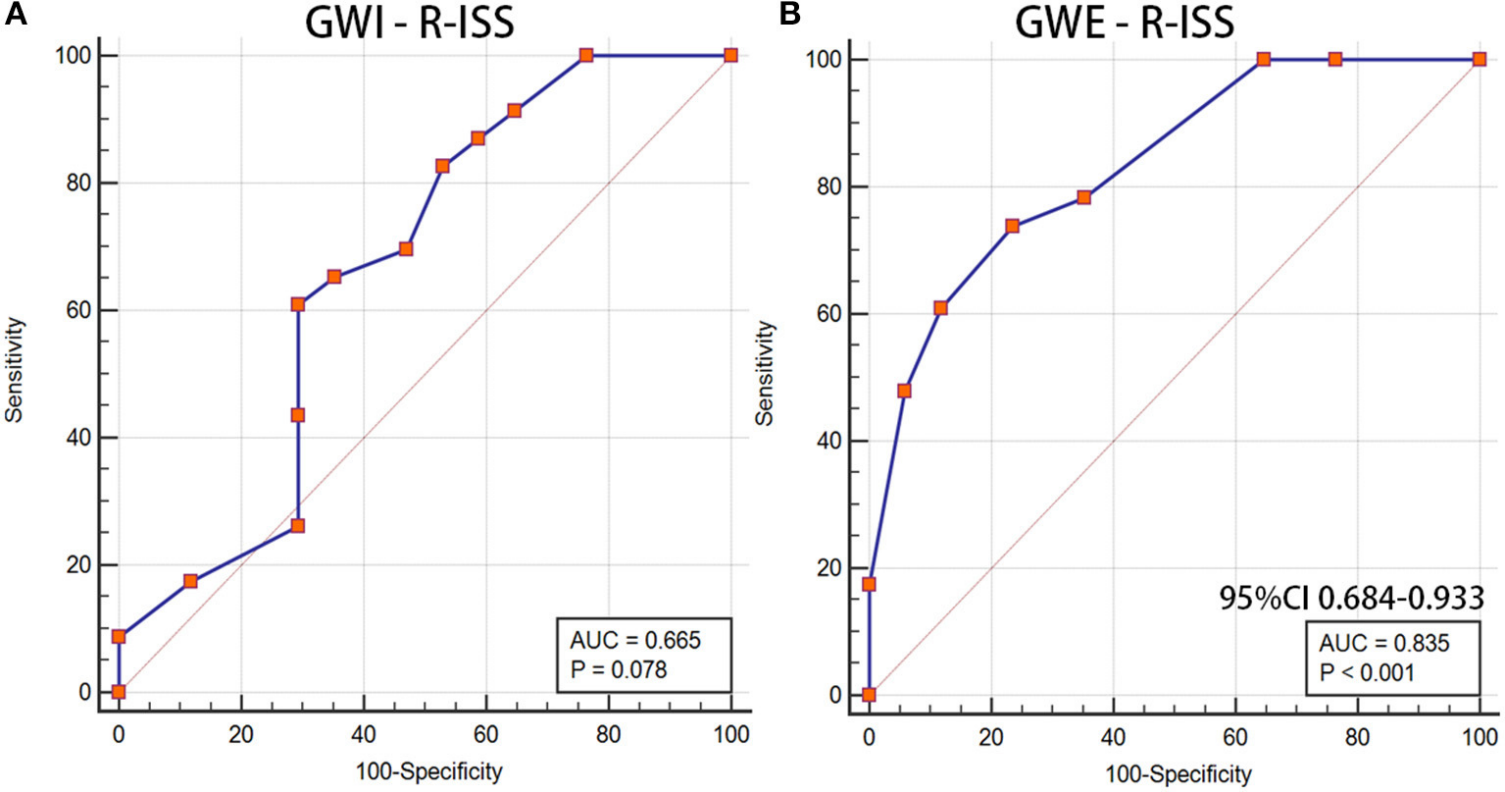
GWI and GWE as a reference to assess the R-ISS of patients with MM. **(A)** LV GWI, **(B)** LV GWE. GWI, global work index; GWE, global work efficiency; R-ISS, Revised International Staging System; MM, multiple myeloma; LV GWI, left ventricular GWI; LV GWE, left ventricular GWE.

Furthermore, we analyzed the diagnostic value of MW in the MM stage. Patients were divided into <III stages and ≥III stages according to the R-ISS. Significant differences were found between the R-ISS < III stages and the R-ISS ≥ III stages group in GWI (1,777.29 ± 458.47 vs. 1,487.52 ± 268.65, *p* = 0.016) and GWE (92.29 ± 3.08 vs. 87.65 ± 3.23, *p* < 0.001), while there was no difference in GLS (17.64 ± 3.06 vs. 16.15 ± 2.87, *p* = 0.122). The diagnostic value of GWI and GWE in the R-ISS is shown in Figure 4. The AUC of GWE for the diagnosis of the R-ISS was 0.835 (95% CI: 0.684–0.933, *p* < 0.05). However, GWI had no diagnostic value for the R-ISS (*p* > 0.05).

### Risk Factors for CAEs in Patients With MM Treated With Bortezomib-Based Therapy

Cardiac adverse events were followed-up after 6 months in patients with MM treated with bortezomib-based therapy. One of these patients had lower extremity edema, one patient had syncope and prolonged RR interval, one patient had new-onset atrial fibrillation, one patient had a decreased LVEF, and three patients had significantly elevated TnT and NT-proBNP levels. Then, patients were divided into the two groups: with or without CAEs. The univariable and multivariable logistic regression models were used to analyze the risk factors of CAEs in patients with MM and the results are shown in Table 5. Sex, age, BMI, course, hypertension, and GWI were not incorporated into the univariable logistic regression model (*p* > 0.1). The multivariable model showed that arrhythmia, Lg_dFLC_, and GWE were independent risk factors for CAEs.

**Table 5:** The multivariable regression analyses of risk factors contributing to CAEs in patients with MM.

	**Univariable analyses**		**Multivariable analyses**	
	**OR [95% CI]**	***P*-value**	**OR [95% CI]**	***P*-value**
Arrhythmia	25.00 [3.30, 189.26]	0.001	10.346 [1.04, 102.75]	0.046
Lg _dFLC_, ng/ml	124.88 [1.59, 9792.82]	<0.001	124.88 [1.59, 9792.82]	<0.001
Lg _Tn−T_, ng/ml	2.75 [0.81, 9.40]	0.094	NA	0.308
Lg _NT−proBNP_, ng/ml	2.71 [1.02, 7.21]	0.025	NA	0.245
GLS, %	0.69 [0.51, 0.95]	0.005	NA	0.507
GWE, %	0.57 [0.38, 0.86]	<0.001	0.60 [0.38, 0.95]	0.006
LVMI, g/m^2^	1.06 [1.00, 1.13]	0.063	NA	0.538

*MM, multiple myeloma; dFLC, difference between involved and uninvolved free light chain; TnT, troponin T; BNP, brain natriuretic peptide; GLS, global longitudinal strain; LVMI, LV mass index; GWE, global work efficiency; NA, not incorporating in the multivariable model*.

### Predictive Value of the Echocardiographic Parameters on CAEs in Patients With MM

**Figure 5:**
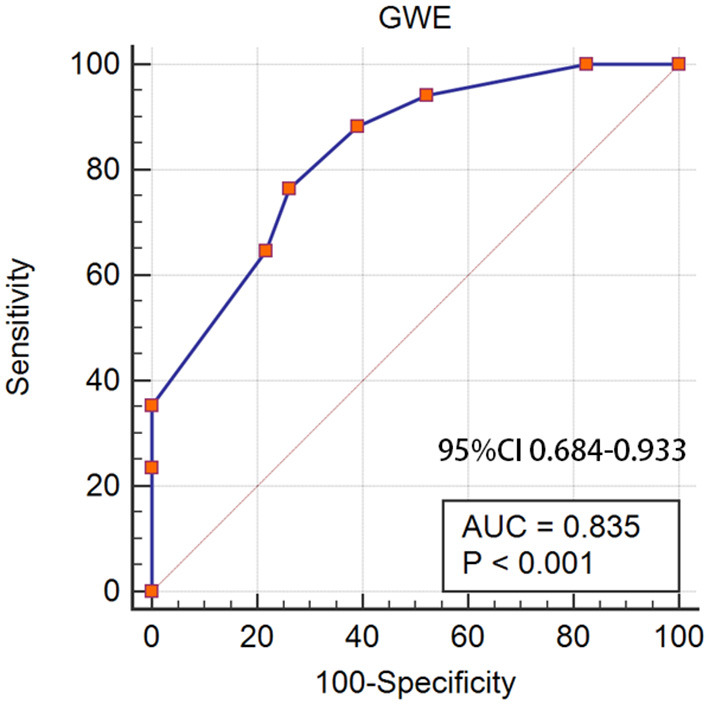
GWE as a prognostic factor of CAEs in patients with MM. GWE, global work efficiency; CAEs, cardiac adverse events; MM, multiple myeloma.

The role of GWE was explored as the prognostic factor of CAEs. A significant difference was found between the CAEs group and the non-CAEs group in GWE (90.55 ± 3.50 vs. 85.29 ± 2.56, *p* = 0.001). The AUC of GWE was 0.896 (95% CI: 0.758–0.970, *p* < 0.05), demonstrating its potential predictive value for CAEs in patients with MM (Figure 5).

## Discussion

The main findings in this study were as follows. First, LV GWI, GWE, and GLS were lower in the thick wall group of patients with MM than in the normal wall group and controls. Cardiac segmental WI in patients with MM showed an apical sparing pattern. Second, GWE had a diagnostic value for disease severity based on the R-ISS. Finally, Lg_dFLC_, arrhythmia, and GWE were the independent risk factor of CAEs and GWE might have a predictive value in patients with MM treated with bortezomib-based therapy for 6 months.

Multiple myeloma is a hematological malignant disease associated with cardiac involvement. Its mechanism may be related to multiple factors, such as amyloidosis, myeloma cell infiltration, hypercalcemia, hyperviscosity, and anemia. Severe cardiovascular complications often occur in the terminal stage, when the disease progresses rapidly, with a high fatality rate (16). Based on routine echocardiography, MM mainly manifests as biventricular hypertrophy, valve thickening, regurgitation, ventricle shrinkage, atrial dilation, increased LV end-diastolic pressure, RV systolic pressure, etc. Myocardial “granule sparkle” is a characteristic manifestation of cardiac amyloidosis in patients with MM, with no specific diagnosis. The LVEF is often in a normal range in the early stage of MM. Reduced LVEF is often associated with the advanced stage (17). Previous studies showed that LV GLS was more sensitive than conventional ultrasound (18).

However, the strain does not determine the effect of afterload pressure, which is higher during late systole lengthening than postsystolic shortening (12). Afterload may result in a reduced LV GLS. Noninvasive LV PSL analysis integrates LS by speckle-tracking analysis with blood pressure measured by mercury sphygmomanometer to estimate MW, which is a new echocardiographic method to evaluate LV function (19). MW measurements have already been applied in various cardiac conditions (20, 21). LVMWI is measured during the entire cardiac cycle, whereas LV GLS only reflects the peak systolic strain (22). Regional WI had a higher sensitivity (81 vs. 78%, *p* < 0.5) and even superior specificity (82 vs. 65%, *p* < 0.5) compared with regional strain to identify acute coronary artery occlusion in patients with non-ST-segment elevation myocardial infarction (23).

For patients with MM with preserved LVEF, LV GWI, GWE, and GLS were lower in the thick wall group than in the normal wall group and controls. There was no difference in GWI and GLS between the normal wall group and controls. However, GWE was lower in the normal wall group compared with controls. LV GWI, GWE, and GLS detected subtle systolic dysfunction in the thick wall group, with GWE showing significant differences. Wall thickening and remodeling of the left ventricle are also correlated with early left ventricular dysfunction.

Significant differences were found in GWI and GWE between patients with MM in the R-ISS stages <III and ≥III. However, only GWE had a diagnostic value for disease severity based on the R-ISS. A lower GWE acted as a predictive value and was the independent factor of CAEs after a 6-month follow-up. GWE included the assessment of constructive work, wasted work, and their contribution to LV ejection, while GWI only measured the total MW. GWE measuring is, therefore, a method for quantifying the work done by the ventricle and contributes to LV ejection. It could also represent a measure of efficient contractility provided that the myocardium is viable.

For segmental changes, no significant difference was found in WI apical among the three groups, showing an apical sparing pattern. Several studies showed apical sparing of LS in patients with MM by speckle-tracking echocardiography (24, 25), which is consistent with this study. Mean LV basal strain is an independent predictor of cardiac and overall deaths (26). Relative sparing in the LV apex may be related to less amyloid deposition occurring in the apex than the base. It is highly sensitive and specific for the diagnosis of cardiac injury in patients with MM (27).

Cardiovascular toxicities are common in patients with MM, which is always lack of specified predictors (28). A meta-analysis of CAEs in patients with MM treated with bortezomib showed an incidence of 4.3% (95% CI: 2.8–6.6%) (29). Currently, specific and effective therapy for cardiovascular toxicities in MM patients is still lacking. Though the angiotensin antagonists, statins, beta-blockers, and nutraceuticals are now under investigation, no clinically significant efficacy was observed so far. Quagliariello et al. (30) found that Empagliflozin, a sodium-glucose cotransporter 2 (SGLT-2) inhibitor, exerted anti-inflammatory and cardioprotective effects in Doxorubicin-induced cardiotoxicity (30). The majority of CAEs (86%) occur within the first 3 months of therapy (28). We sought to determine the risk and predictors of CAEs from 6 months follow-up and found that the rate of arrhythmia, Lg_dFLC_, and GWE were the independent risk factors of CAEs in treated patients with MM. A previous study showed that patients who had a history of arrhythmia were likely to be attacked again after therapy and the median length of hospital stay was prolonged (31). The dFLC also exhibits predictors for clinical treatment response and an association with both cardiac involvement and disease progression (32). This study proved that GWE may be an alternative to predict CAEs in patients with MM. Overall, CAEs risk assessment by cardiac reserve capability in a timely and effective manner helped to reduce the mortality and hospital readmission rate of patients with MM.

This study has several limitations. First, this study had a small sample size and a short follow-up period. However, it should be borne in mind that MM is a rare disease. The age-standardized incidence rate of MM was 1.1/1,000 in 2018. On the other hand, the strength of our results lies in the fact that we recruited subjects without medication. Second, part of follow-up data obtained by telephone could be biased; most patients with immunodeficiency chose to stay at home rather than travel to the hospital due to the impact of coronavirus disease 2019 (COVID-19). Further prospective studies with a larger size sample are needed.

## Conclusion

MM patients with preserved EF had subclinical left ventricular systolic dysfunction, which was worse in the thick wall group. GWI presented an “apical sparing” pattern in patients with MM. A lower LV GWE may have a diagnostic and predictive value for disease severity and CAEs in patients with MM treated with bortezomib-based therapy for 6 months.

## Data Availability Statement

The raw data supporting the conclusions of this article will be made available by the authors, without undue reservation.

## Ethics Statement

The studies involving human participants were reviewed and approved by Biomedical Research Ethics Committee of West China Hospital, Sichuan University. The patients/participants provided their written informed consent to participate in this study. Written informed consent was obtained from the individual(s) for the publication of any potentially identifiable images or data included in this article.

## Author Contributions

ZL designed the study and wrote the manuscript. LZ, YX, ZT, QLi, and SL collected the data and generated the database. FW, QLu, and ML analyzed and interpreted the data. HH and TN supervised the study. All authors critically revised the manuscript for important intellectual content.

## Funding

HH is funded by Key Research and Development Programs of the Science and Technology Department of Sichuan Province (Grant no. 2019YFS0414).

## Conflict of Interest

The authors declare that the research was conducted in the absence of any commercial or financial relationships that could be construed as a potential conflict of interest.

## Publisher's Note

All claims expressed in this article are solely those of the authors and do not necessarily represent those of their affiliated organizations, or those of the publisher, the editors and the reviewers. Any product that may be evaluated in this article, or claim that may be made by its manufacturer, is not guaranteed or endorsed by the publisher.
